# Comparison of virus neutralization activity and results of 10 different anti-SARS-CoV-2 serological tests in COVID-19 recovered plasma donors

**DOI:** 10.1016/j.plabm.2021.e00222

**Published:** 2021-04-20

**Authors:** Zsófia Szabó, Tamás Szabó, Kornélia Bodó, Gábor Kemenesi, Fanni Földes, Katalin Kristóf, Eszter Barabás, Barna Vásárhelyi, Zoltán Prohászka, Eszter Fodor, Ferenc Jakab, Timea Berki, Zsombor Lacza

**Affiliations:** a Department of Laboratory Medicine, Semmelweis University, Budapest, Hungary; b Institute of Developmental Immunology, Medical University of Innsbruck, Innsbruck, Austria; c Department of Immunology and Biotechnology, Clinical Centre, University of Pécs, Pécs, Hungary; d National Laboratory of Virology, BSL-4 Laboratory, Szentágothai Research Centre, University of Pécs, Pécs, Hungary; e Institute of Biology, Faculty of Sciences, University of Pécs, 7622, Pécs, Hungary; f Department of Internal Medicine and Hematology, Semmelweis University, Budapest and Research Group for Immunology and Hematology, Semmelweis University- Eötvös Loránd Research Network (Office for Supported Research Groups), Budapest, Hungary; g Institute of Sports and Health Sciences, University of Physical Education, 1112, Budapest, Hungary

**Keywords:** SARS-CoV-2, COVID-19, Correlate of protection, Serological test, Antibody response, Neutralization, SARS-CoV-2, Severe acute respiratory syndrome coronavirus 2, COVID-19, coronavirus disease 2019, AUC, area under the curve, ROC, receiver operating characteristic, ECDC, European Centre for Disease Prevention and Control, NAbs, neutralizing antibodies, VNT, virus neutralization test, NC, Nucleocapsid, S1, Spike protein 1, S2, Spike protein 2, OD, optical density

## Abstract

Serological testing is a tool to predict protection against later infection. This potential heavily relies on antibody levels showing acceptable agreement with gold standard virus neutralization tests. The aim of our study was to investigate diagnostic value of the available serological tests in terms of predicting virus neutralizing activity of serum samples drawn 5–7 weeks after onset of symptoms from 101 donors with a history of COVID-19.

Immune responses against Receptor Binding Domain (RBD), Spike1 and 2 proteins and Nucleocapsid antigens were measured by various ELISA tests. Neutralizing antibody activity in serum samples was assessed by a cell-based virus neutralization test.

Spearman correlation coefficients between serological and neutralization results ranged from 0.41 to 0.91 indicating moderate to strong correlation between ELISA test results and virus neutralization. The sensitivity and specificity of ELISA tests in the prediction of neutralization were 35–100% and 35–90% respectively. No clear cut off levels can be established that would reliably indicate neutralization activity. For some tests, however, a value below which the sample is not expected to neutralize can be established. Our data suggests that several of the ELISA kits tested may be suitable for epidemiological surveys 1–2 months after the infection, estimating whether a person may have recently exposed to the virus. Sensitivities considerably superseding specificity at the cut-off values proposed by the manufacturers suggest greater potential in the identification of insufficient antibody responses than in confirming protection. Nevertheless, the former might be important in assessing response to vaccination and characterizing therapeutic plasma preparations.

## Highlights

• SARS-CoV-2 antibody levels 5-7 weeks after the onset of COVID show moderate to strong correlation with virus neutralization.
• Tests based on the use of S1, nucleocapsid or RBD antigens performed similarly in predicting antibody neutralization.
• None of the examined serological tests could safely identify individuals protected against a later SARS-CoV-2 infection.
• The products assessed might still provide important epidemiological information.
• Serological tests might still have potential in screening donors for therapeutic plasma products or vaccinated individuals.

## 1 Background

Little is known about the extent and duration of immunity induced by SARS-CoV-2 infection. Specific antibody response to SARS-CoV-2 infection has not been sufficiently mapped and antibodies of clinical significance are not well characterized yet [1]. Testing antibody levels may help establish or support the diagnosis in some cases [2,3] or may play a role in the prediction of clinical course [1,4]. A further expectation from routine laboratories is to assess the status of protection against reinfection or new infection by SARS-CoV-2 after vaccination in the future. Literature refers to the laboratory parameter indicating protection due to infection or vaccination as a “correlate” or “surrogate” parameter [5]. Although the results of several reports suggest that cellular immune response plays a major role in the development of protection [6], serological tests are more easily accessible to routine laboratories. Therefore, there is a need to clarify the diagnostic value of SARS-CoV-2 serological tests.

In case of SARS-CoV-2, the virus mainly enters the host cell by binding to the ACE2 receptor and this process can be blocked by the neutralizing antibody provided that it binds to the receptor binding domain (RBD) of S1 protein [7]. Neutralization assays usually are not feasible for routine laboratories. Therefore, specific antibody titers are an intriguing alternative to be used as “correlate” or “surrogate” of protection if they correlate with the result of neutralization test. Assessing protection is expected to be of great health and economic importance in the future as well. Examining the effectiveness of vaccination specific IgG testing is also likely to be one of the most important laboratory tests.

Furthermore, these results are important for the application of plasma therapy, which is a widely studied procedure for the treatment of COVID-19 patients. Although promising, convalescent plasma has not yet been shown to be safe and effective as a routine treatment for COVID-19 [8]. Examination of the antibody content of donor samples yieldsimportant information to better understand and improve the efficacy and safety of convalescent plasma therapy.

There are various commercially available tests [2] which detect antibodies with different antigen specificities and, probably, with different functionality. Our aim was to analyze and compare antibody levels measured by different products to the results of a neutralizing activity assay in sera of patients recovered from COVID-19. Linking neutralization activity with serological test result helps to better characterize their diagnostic value, and, also, might extend their usage to a variety of scenarios: establishment of diagnosis, confirming protection against infection, conducting epidemiological surveys or characterization of plasma preparations of therapeutic purposed.

## 2 Patients and methods

### 2.1 Patients and blood samples

The study was performed under IRB approval (number IV/3457/2-2020-EKU, ClinicalTrials.gov Identifier: NCT04345679). Sera (n ​= ​101) obtained from individuals recovered from SARS-CoV-2-infection confirmed by real-time RT-PCR (n ​= ​30), or recovered individuals diagnosed as probable COVID patients based on clinical symptoms plus the presence of SARS-CoV-2- specific antibodies (n ​= ​71) according to ECDC case definition (29.05.2020) [9]. 26% of patients suffered from moderate or severe illness according to WHO classification (27.05.2020) [10] and 74% had mild symptoms. Due to the limited volume of some samples, not all measurements could be performed from all samples.

### 2.2 Measurement of virus neutralizing titer

VeroE6 cells (African green monkey kidney cell line) were grown in Dulbecco’s modified eagle medium DMEM (Lonza), supplemented with 2% fetal bovine serum (FBS) (EuroClone), 1% Penicillin–Streptomycin (Lonza), and maintained at 37 ​°C in a humidified atmosphere containing with 5% CO_2_. The SARS-CoV2 isolate of the Szentágothai Research Centre Biosafety Laboratory Level 4 was used in these experiments. The viral titer of the stock was previously determined by TCID50 assay.

During the neutralization assay procedure, 50 ​μl two-fold serially diluted and heat-treated (56 ​°C 30 ​min) sera were incubated with 50 ​μl DMEM containing 100 TCID50 of SARS-CoV-2 for 1 ​h at 37 ​°C in 96-well microtiter plates. Positive control wells were not containing serum samples, only 100 TCID50 of SARS-CoV-2. Negative cell control wells were not containing serum samples, only DMEM. Confluent VeroE6 cells were infected with 100 ​μl two-fold serially diluted neutralized virus for 30 ​min. After the infection, 150 ​μl sustaining media was added to the cells and incubated for 3 days at 37 ​°C 5% CO2. The serum neutralization titer was determined by calculating the highest dilution of sera that prevents infection of 50% of replicate inoculations. Three replicates were used from diluted serum samples and controls.

### 2.3 ELISA assays

SARS-CoV-2 specific antibodies were analyzed using commercially available antibody detection kits according to the test descriptions. Generic Assay (GA) and GenScript ELISA tests were performed at Semmelweis University, Department of Laboratory Medicine. GA assay (Cat No. 3940) measures specific IgG against Spike1 and 2 proteins (S1 and S2) and Nucleocapsid (NC) specific antibodies in three separate, but parallel wells of the same plate. Thus, it gives well-comparable results for antibodies specific for the three different antigens. We followed the manufacturer’s recommendations for semi-quantitative characterization and determination of positivity (binding index <1 U/ml negative, 1.0–1.2 U/ml borderline, >1.2 positive). The GenScript Surrogate Virus Neutralization Test Kit (Cat No. L00847) can detect neutralizing antibodies (NAbs) against SARS-CoV-2 in an isotype independent manner using purified receptor-binding domain (RBD) and the host cell receptor ACE2. This RBD–ACE2 interaction can be neutralized by specific NAbs in patient sera [11]. Inhibition was calculated due to the manufacturer’s recommendations (<20% negative, ≥20% positive).

Indirect ELISA test of 5 different manufacturers has been compared for the presence of anti-SARS-CoV-2 IgG, IgA and IgM antibodies against Spike and/or Nucleocapsid proteins at University of Pécs, Department of Immunology and Biotechnology. Mikrogen ELISA for Nucleocapsid IgG (Cat No. 7304) (<20 U/ml negative, 20–24 U/ml borderline, >24 U/ml positive) Euroimmun ELISA for Spike1 IgG and IgA (Cat. No. EI 2606–9601 A and G) (0.8–1.1 U/ml: borderline, >1,1 U/ml positive), Dia.Pro ELISA for recombinant Spike and Nucleocapsid IgG/IgA/IgM (0.9–1.1 U/ml: borderline, >1,1 U/ml positive) and Vircell ELISA for recombinant Spike and Nucleocapsid proteins IgG (Cat No. G1032) (4,0–6,0 U/ml: borderline, >6,0 U/ml: positive) and Vircell IgA ​+ ​IgM were compared in a single measurement (Cat No. MA 1032) (6,0–8,0 U/ml: borderline, >8,0 U/ml: positive). For convenience Table 1 shows the antigens used by each assay and also the isotype of detected serum antibodies.

**Table 1:** **Test statistics of different SARS-CoV-2 ELISA kits.** The table shows the characteristics of studied tests, including antigens used by each assay as well as isotype of detected antigens and all statistical parameters using manufacturer-provided cut-off values.

	**Manufacturer**	**notation**	**antibody**	**n**	**Sensitivity**	**Specificity**	**Cohen’s kappa r**	**Spearman r**	**AUC**
1	Generic Assay	GA_S1	S1 IgG	58	100.0	35.5	0.34	0.65	0.87
		GA_S2	S2 IgG	61	34.6	84.8	0.19	0.41	0.73
		GA_NC	Nucleocapsid IgG	60	100.0	36.4	0.34	0.62	0.82
2	Dia.Pro	Diapro_IgG	Spike and Nucleocapsid IgG	70	100.0	66.7	0.45	0.67	0.84
3	Dia.Pro	Diapro_IgA	Spike and Nucleocapsid IgA	70	73.3	92.5	0.61	0.75	0.90
4	Dia.Pro	Diapro_IgM	Spike and Nucleocapsid IgM	70	64.3	90.6	0.50	0.61	0.78
5	Euroimmun	Euroimmun_IgG	S1 IgG	70	100.0	74.1	0.57	0.70	0.88
6	Euroimmun	Euroimmun_IgA	S1 IgA	70	100.0	59.3	0.40	0.67	0.86
7	Mikrogen	Mikrogen_IgG	Nucleocapsid IgG	70	93.8	75.9	0.55	0.67	0.84
8	Vircell	Vircell_IgG	Spike and Nucleocapsid IgM	70	100.0	69.8	0.50	0.59	0.78
9	Vircell	Vircell_IgAM	Spike and Nucleocapsid IgG	70	93.3	40.5	0.17	0.58	0.80
10	GenScript	GenScript_RBD	RBD isotype independent	67	100.0	38.5	0.34	0.72	0.92

### 2.4 Statistical analysis

Statistical analyses were performed using Graphpad Prism software, version 7.0 and the Python (version 3.8) libraries Pandas (version 1.0.4), Seaborn (version 0.10.1) and SciKit Learn (version 0.23.1).

Spearman correlation coefficients were used to evaluate correlation between ELISA tests and virus neutralization. The AUC of ROC curves, as well as Sensitivity, Values were to characterize performance of the ELISA tests as surrogate markers for neutralization.

## 3 Results

### 3.1 Correlations of antibody responses and neutralizing activity

In order to assess the diagnostic value of various commercially available ELISA tests and to investigate the specific antibody profile of individuals recovered from COVID-19, we evaluated SARS-CoV-2 specific antibody responses in serum samples collected from volunteers offering convalescent plasma for therapeutic application. Quantitative results of ELISA tests (101 samples from 101 patients) were plotted against the reciprocal neutralizing titer. Fig. 1a. Median antibody levels increased with increasing neutralizing activity, except for GA S2. Fig. 1b. In individuals with negative virus neutralization tests (i.e. under 1:10), antibody levels vary greatly (see numeric data on Fig. 1c). It is important to note that in some subjects high antibody levels could also be observed in the absence of neutralization. Thus, no threshold can be established above which neutralization activity is clearly present. On the other hand, in case of GenScript anti-RBD test a clear cut off can be established (40%) below which the sample is not expected to neutralize Fig. 1a. A similar cut off value for the neutralizing capacity of measured IgG in the different ELISA tests can be proposed: GA NC and S1 IgG: 2 (binding index); Euroimmun IgG: 2 U/ml; Mikrogen IgG 40 U/ml; Vircell IgG: 6 U/ml; Dia.Pro IgG: 2 U/ml where the positive neutralization is not likely.

**Fig. 1a:**
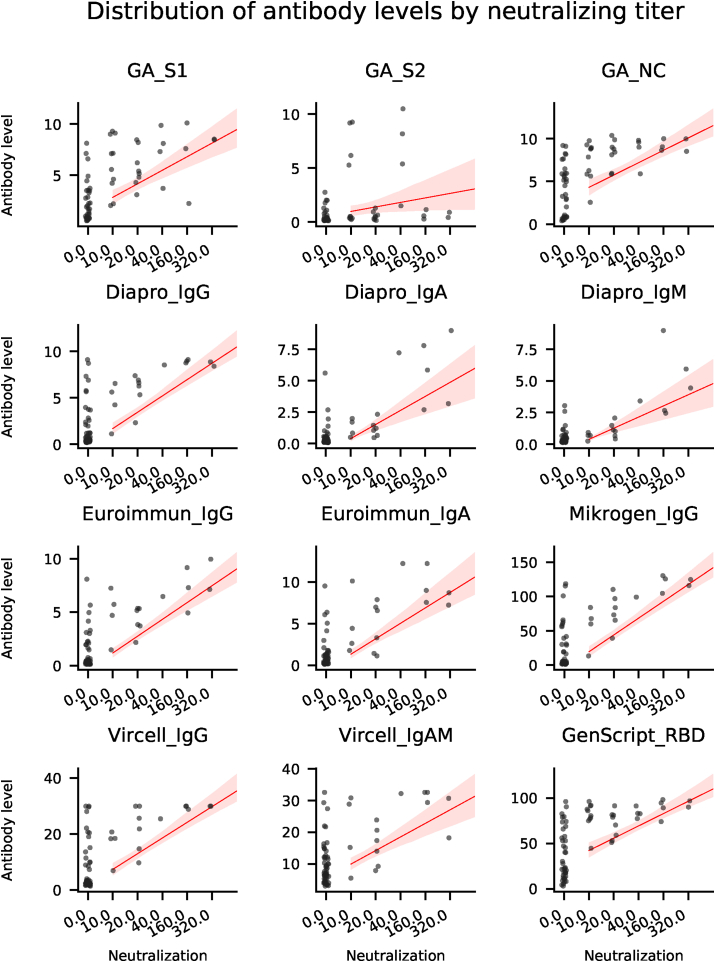
Antibody response against different SARS-CoV-2 antigens. Correlation of SARS-CoV-2 neutralizing antibody titers tested by cell-based (Vero6 cell) virus neutralization assay to antibodies measured by commercial ELISA tests. The serum neutralization activity titer was determined by calculating the highest dilution of sera that prevents infection of 50% of replicate inoculations. X axis indicates 1/neutralization titer (dilution) on ordinal scale. Y axis indicates antibody levels that were measured by commercial ELISA tests. Regression lines (in red) with shaded confidence intervals were calculated using standard settings in the Python package Seaborn. Antibody levels were determined according to the prescription of manufacturers. For GA (n ​= ​61), „Binding index” was calculated as following: OD of sample/OD Co (Co: cut-off OD). GenScript „Virus Neutralization Test” with RBD antigen (n ​= ​67) offers a calculated „Inhibition” readout: (1 - OD of sample/OD of negative control) ​× ​100%. For the further ELISA tests, the antibody levels were also determined according to the manufacturer’s instructions, calculated from the OD ratios (n ​= ​70). (Mikrogen ELISA for NC IgG, Euroimmun ELISA for S1 IgG and IgA, Dia.Pro ELISA for recombinant Spike and NC IgG/IgA/IgM, Vircell ELISA for recombinant Spike and NC proteins IgG and Vircell IgA ​+ ​IgM in a single measurement.). (For interpretation of the references to colour in this figure legend, the reader is referred to the Web version of this article.)

**Fig. 1b:**
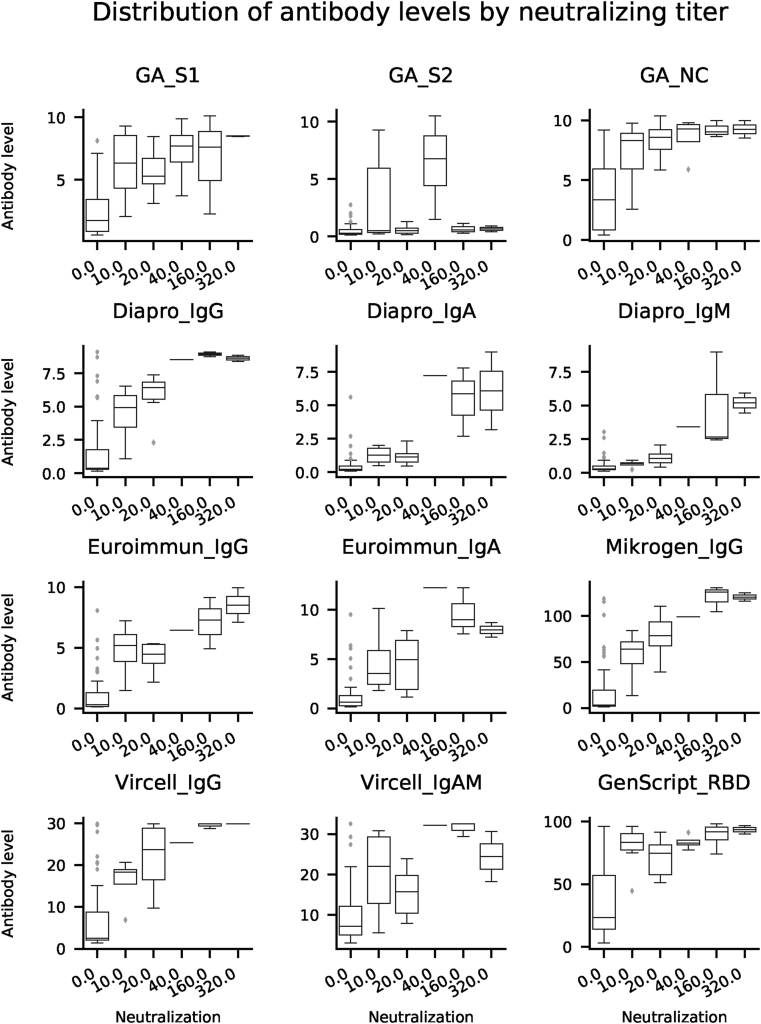
Distribution of antibody levels by neutralizing titer. Median values, upper and lower quartiles of antibody levels as a function of virus neutralization activity are plotted for the same samples as in Fig. A1. Box plots were generated using default parameters in the Python package Seaborn.

**Fig. 1c:**
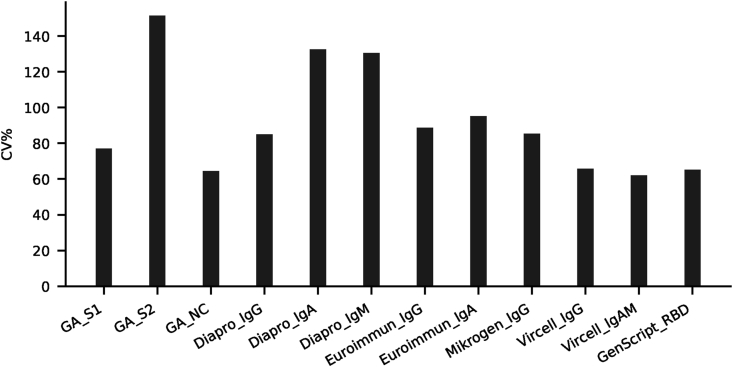
Coefficient of variation of antibody levels in non-neutralizing samples. For the same samples shown above on Figs. A1 and A2. Coefficient of variation was calculated as 100 ∗ Standard Deviation/Mean using numpy.

**Fig. 2:**
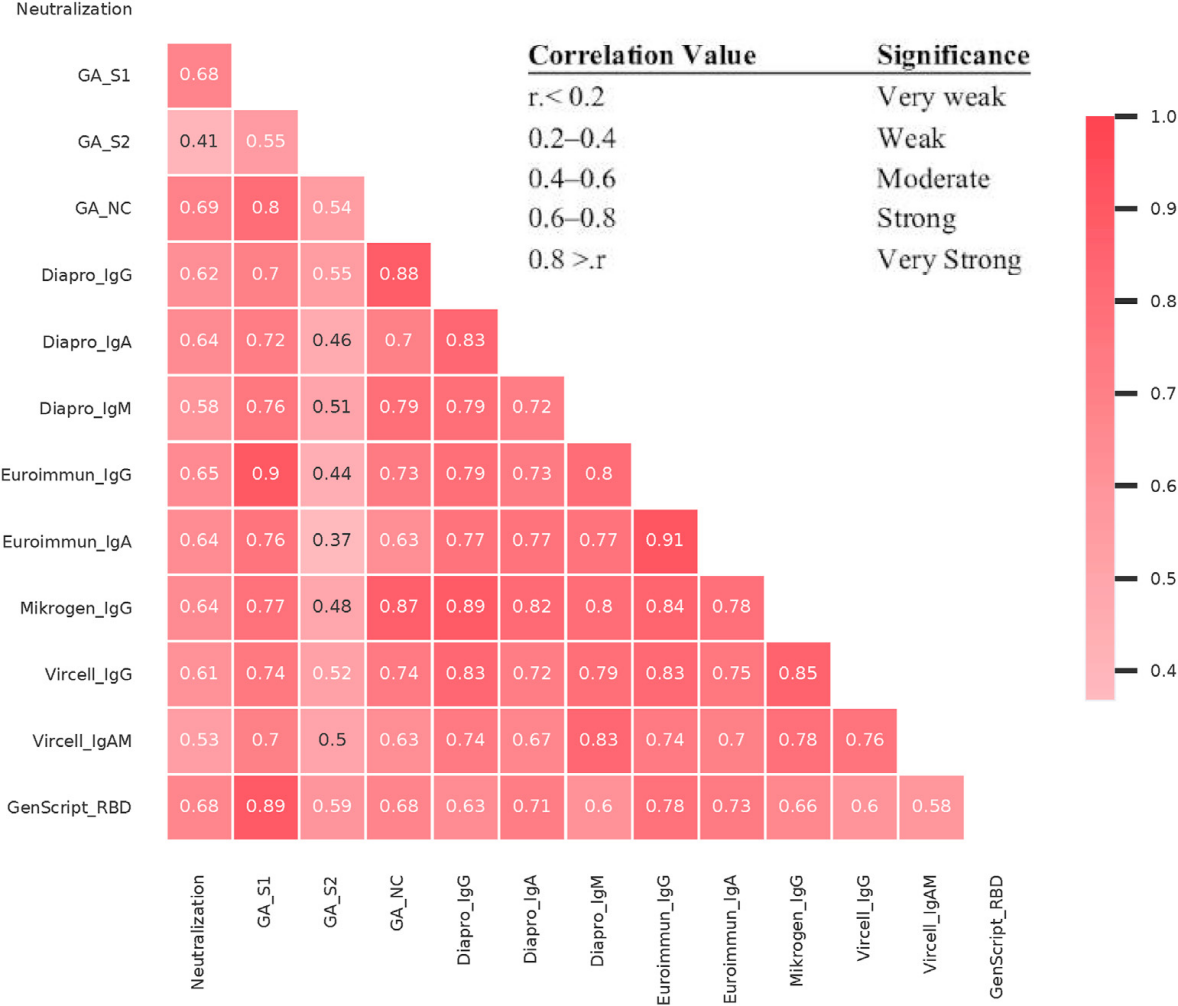
Spearman’s correlation coefficient among serological tests and virus neutralization activity. The correlations among antibody levels against different viral antigens and virus neutralization activity were analyzed and summarized by the Python library Pandas and visualized by Seaborn. Data for the same samples as shown on earlier figures.

Spearman correlation coefficients (Fig. 2.) indicate moderate to very strong correlation among neutralization assay and ELISA tests. The correlation was strong (r ​≥ ​0.6) in case of GA S1-, and NC IgG tests, GenScript anti-RBD test, Mikrogen NC IgG, Euroimmun S1 IgG/IgA, Dia.Pro S/NC IgG/IgA, Vircell (S/NC) IgG ELISA tests. Test statistics are summarized in Table 1.

**Fig. 3:**
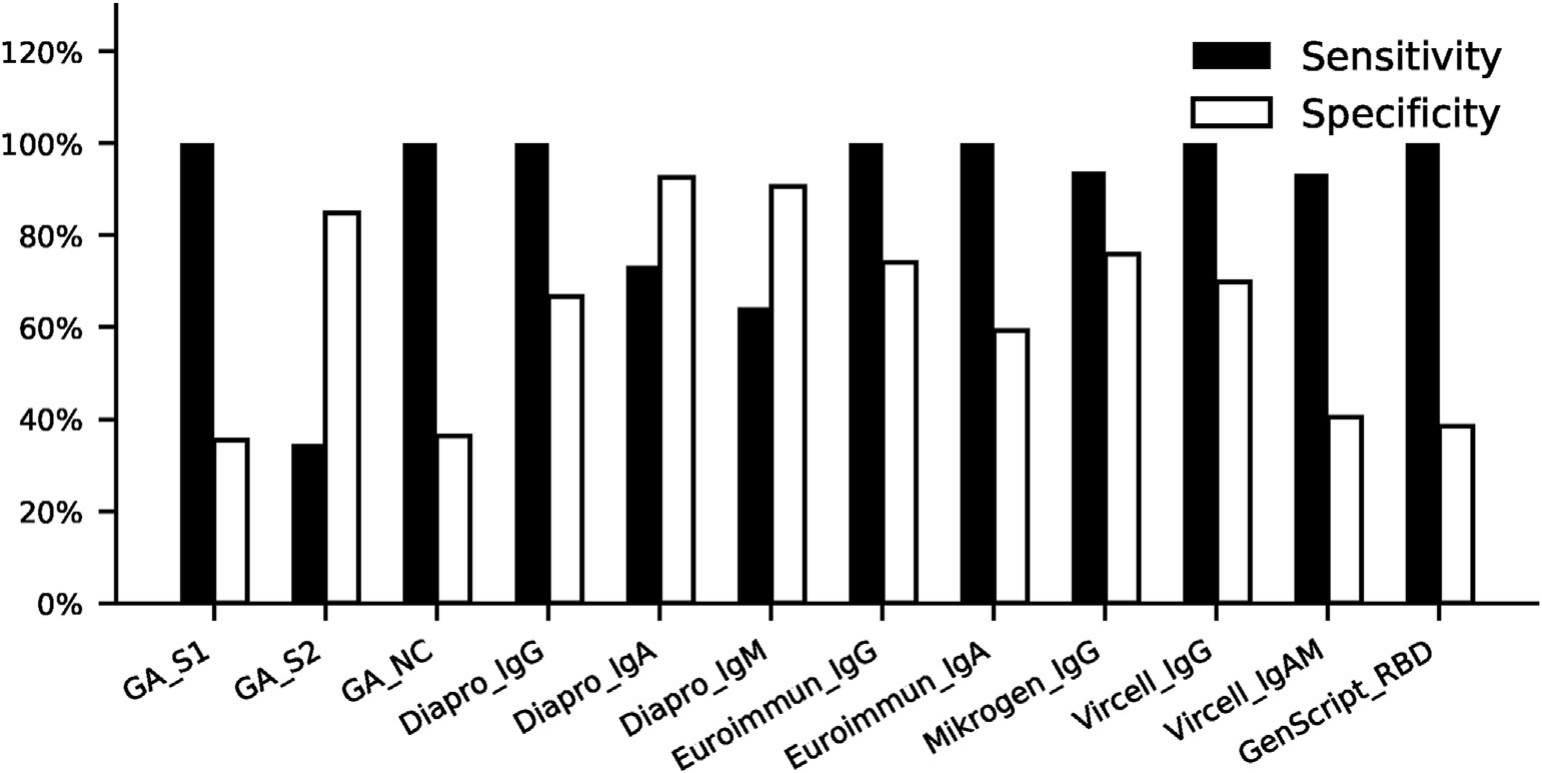
Sensitivity and specificity of ELISA tests for neutralization activity. Neutralization titer <1:10 was considered negative and ≥1:10 was considered positive. Sensitivity and specificity of ELISA tests were calculated for positive neutralization activity.

Sensitivity and specificity values of ELISA tests with respect to neutralization are shown on Fig. 3. The best sensitivities to neutralization (at default cut off values) were observed in case of GA S1 and NC tests, Euroimmun IgG and IgA tests, Dia.Pro IgG, Vircell IgG and Genscript RBD tests. Mikrogen IgG and Vircell IgA and IgM were almost as sensitive as the former.

Agreement among qualitative results of ELISA immunoassays and those of neutralization assay was also analyzed. Neutralization titers which were lower than 1:10, were considered as negative. This cut-off titer is lower than what is usually used in the literature [12,13], but since there was no sample with positive neutralization without at least 3 positive ELISA tests, this seemed to be a reasonable cut-off in our study. The highest dilution of sera that prevents infection of 50% of replicate inoculations and was 1:10 dilution or higher was considered as positive.

**Fig. 4:**
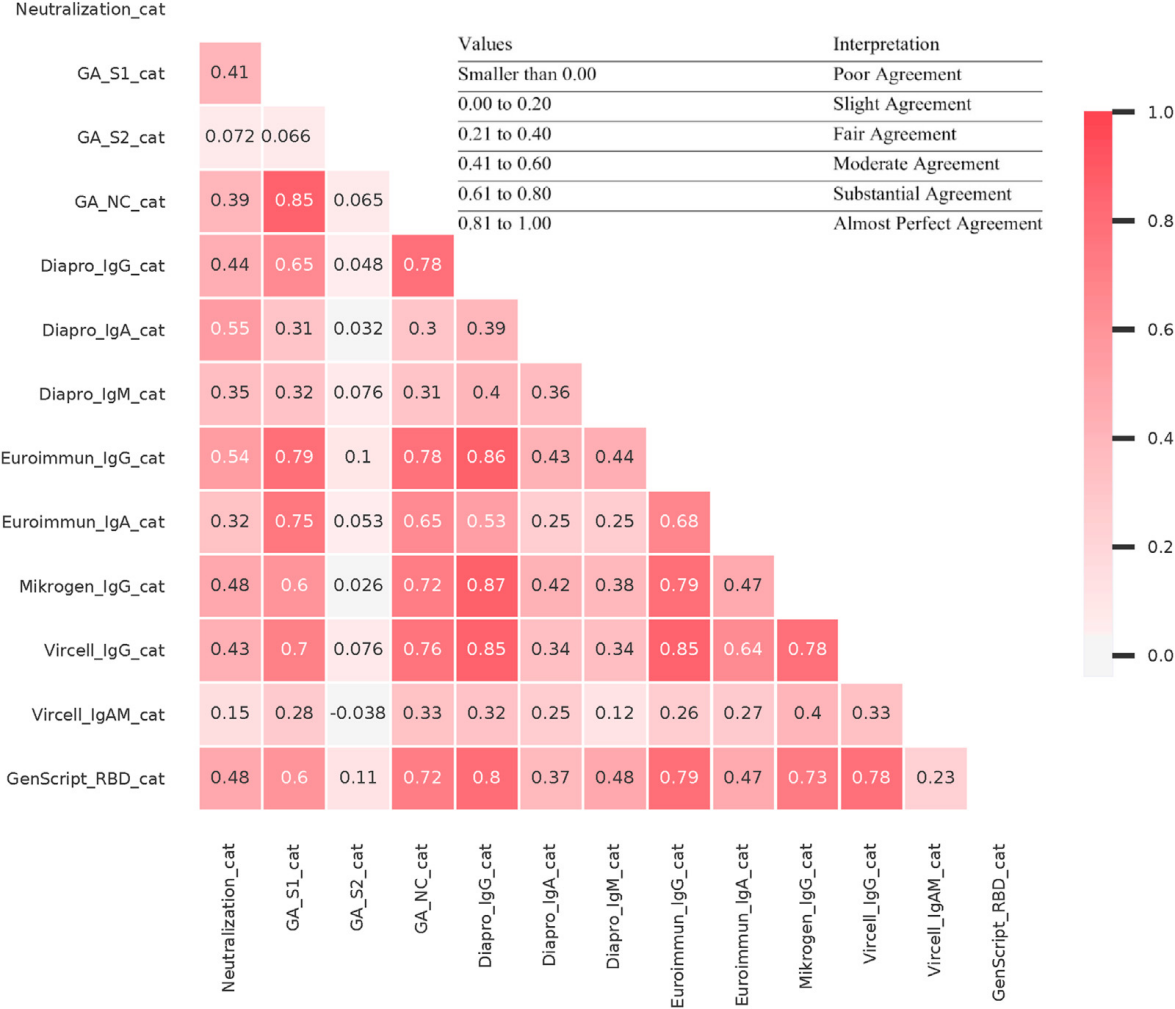
Cohen’s kappa coefficient among serological tests and virus neutralization activity. The degree of agreement among qualitative results of serological tests and virus neutralization activity according to Cohen’s Kappa score. Calculations were done using the Python library Scikit learn.

Concerning neutralization, Cohen’s kappa values for agreement were similar in case of ELISA tests containing S protein antigen or NC (Fig. 4). This suggests that the two major antigens in current serological tests have similar performance and might elicit a shared antibody profile. The range was: 0.17–0.61 which indicates only moderate correlation. The best performing tests were Dia.Pro IgA, Euroimmun IgG and Mikrogen IgG.

**Fig. 5:**
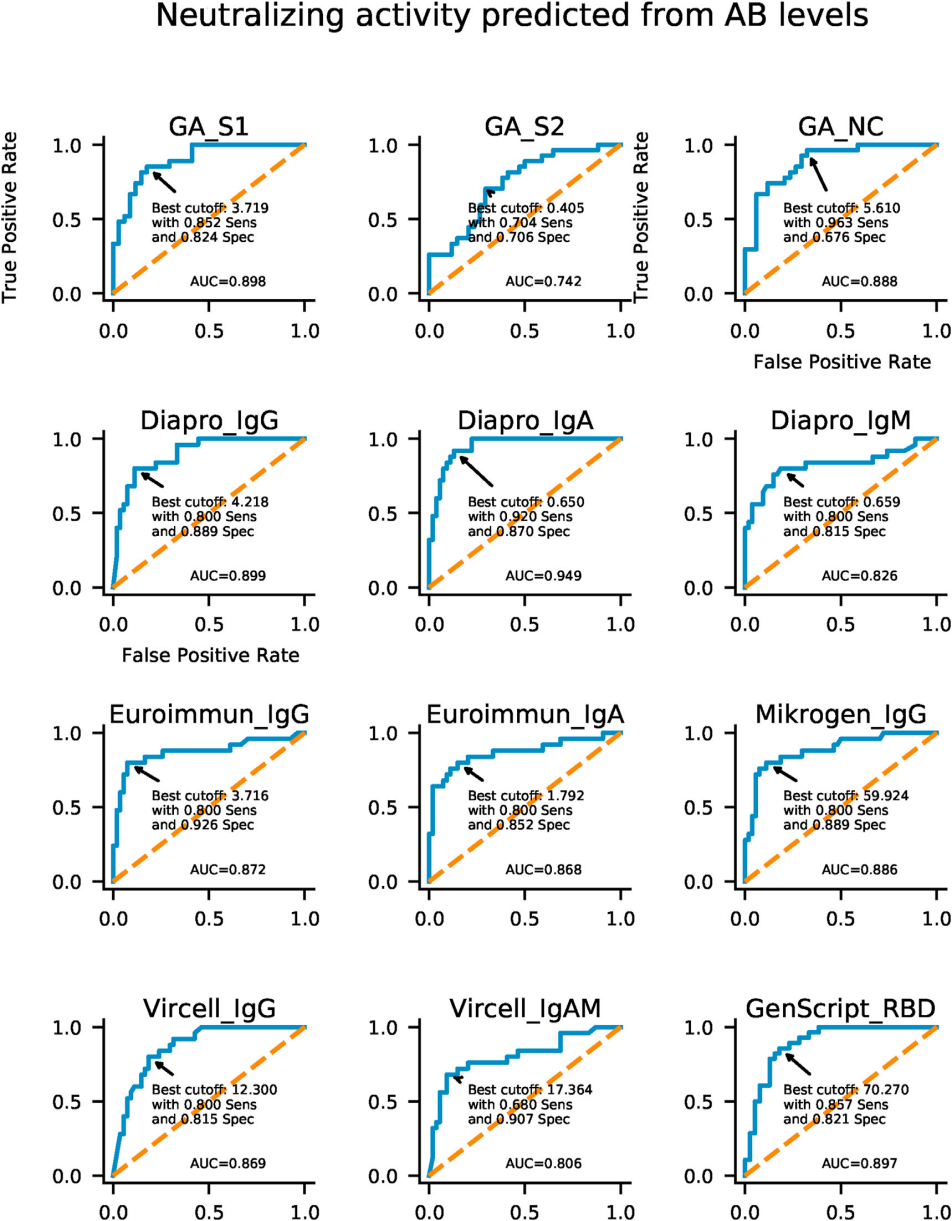
ROC (Receiver operating characteristic) analysis for anti-SARS-CoV-2 antibodies detection. ROC curves show AUC (Area Under the Curve) values from 0.74 to 0.95. Based on ROC curve data, optimal cut off values of antibody levels were evaluated for neutralization activity.

**Fig. 6:**
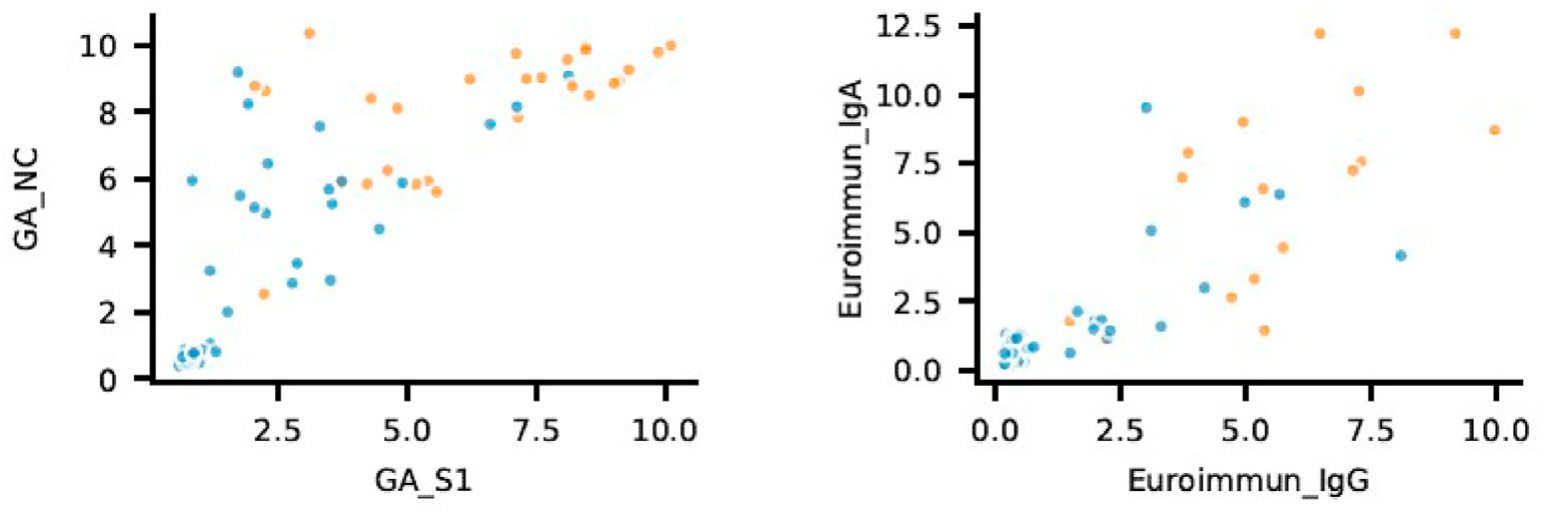
Combination of different tests. Orange dots indicate positive neutralization results; blue dots indicate negative neutralization results. Combination of S1 and NC antigen (e.g.Generic Assay, n ​= ​61) or different isotypes (Euroimmun IgG and IgA, n ​= ​70) are shown as a possible approach to improve predictive value of testing strategy. (For interpretation of the references to colour in this figure legend, the reader is referred to the Web version of this article.)

Cut-off optimization is largely dependent on whether the test is intended to be used for the purpose of positive (specificity being more important) or negative selection (relying more on sensitivity). ROC analysis and cut-off optimization show that cut-off values recommended by manufacturers are already close to optimum specific to the present cohort and there is little space for further optimization towards higher specificities while keeping sensitivity in acceptable range (Fig. 5.). Combination of different tests, like combining S1 with NC antigen (e.g.Generic Assay) or different isotypes (Euroimmun IgG and IgA) can improve predictive potential, as suggested by Fig. 6.

## 4 Discussion

Antibody response to SARS-CoV-2 virus exposure is heterogeneous, and, therefore, the neutralizing effect of antibodies differs [14]. Although neutralization is an important feature, it is resource-intensive to measure [15] and routine clinical diagnostic laboratories do not perform its measurement. A better understanding of its correlation with surrogate markers, like results of ELISA tests, however, might provide a means to overcome this bottleneck in the follow-up of response to infection. Something especially relevant these days have given the current situation of SARS-CoV-2 pandemic.

Spearman correlations between ELISA tests containing S1 and NC antigens and neutralization showed moderate to strong results similar to other studies on convalescent sera [16,17]. The best correlations with neutralization were found for GA NC (0.69), GA S1 (0.68) and GenScript RBD (0.68) tests, suggesting that similar performance can be expected from tests containing any of the above three antigens. None of the serological tests performed well enough to establish protection of an individual based on the current gold standard cellular neutralization assay in a cohort of 101 recovered subjects.

Imperfect performance of tests, and low overall neutralization titers might arise from the study design sampling only 5–7 weeks after the first COVID-19 symptoms. It is possible that at earlier time points, higher antibody levels could have been measured with a better correlation with neutralization activity. The sampling time, approximately a month after recovery, is more relevant information for the long-term protection, however: antibody levels and neutralizing titers already diminishing this early cannot be expected to provide an effective long-term protection. It is also of note that external quality control (Nequas) samples are also taken 30–35 days after recovery of COVID-19 patients. A follow-up of antibody levels would be needed to determine optimal time for sampling. All the more, it is even advisable to extend the study to later time points, since decline of antibody levels may differ for the different antigen specificity.

Although the RBD-containing ELISA was expected to better match neutralization results, the present study is not the only one featuring this controversy. In a recent paper, poor correlation was attributed to the existence of multiple different epitopes on RBD that do not engage in receptor binding [18]. It was also speculated that during the production of the ELISA test neoepitopes may be created or unmasked [18]. Moreover, it is already known that the virus can enter human cells via alternative receptors (e.g. neuropilin-1, DC-SIGN, L-SIGN [[19], [20], [21]]). Anti-RBD test performance might also vary among products of different manufacturers: stronger correlation (r ​= ​0.829) was observed with reagent different from the one used in the present study [13].

Negative and positive agreement among ELISA tests and neutralization in our cohort varied from slight to substantial according to Cohen’s kappa value. This suggests that differences between commercially available SARS-CoV-2 specific antibody ELISA tests are occasionally remarkable.

Clear ELISA cut-off values for reliably predicting neutralization activity could not be determined from ROC curves, and, therefore, our results do not support the usage of ELISA tests as a “correlate” or “surrogate” parameter for neutralization. If the purpose of the test is not screening for protected individuals, but detection of the absence of protection, ELISA tests show great potential in the present cohort. In this scenario, the exact cut-off values should be established on a larger cohort of patients with a larger focus on sensitivity. These cut-off values could significantly differ from those currently recommended by manufacturers, optimized for general testing.

Optimized for higher sensitivity, ELISA tests would offer a high-throughput means to monitor response to vaccination at the population level and identify individuals who might need revaccination. Another example could be screening individuals volunteering to donate convalescent plasma. ELISA tests could be first-pass tests to rule out volunteers not expected to feature sufficient levels of neutralizing antibodies.

Our observations indicate that while there is small difference in the performance of tests using individual antigens (S1, NC and RBD), combining tests featuring different antigens might add to predictive value. A special case would be to combine the results of GA S1 and GA NC in the current study. This approach is different from just adding both antigens to the same well in the ELISA plate. While the latter might improve sensitivity, only the former can improve specificity - the key parameter for the establishment of protection of an individual.

In our study the tests with the highest performance values were GA S1-, and NC IgG tests, GenScript anti-RBD test, Mikrogen IgG, Euroimmun IgG and IgA, Dia.Pro IgG and IgA, Vircell IgG ELISA tests.

Although ELISA tests are already utilized for epidemiological surveys and to individually establish a history of previous infection, their availability makes further extension of use cases appealing. Our observation that anti-SARS-CoV-2 antibody levels in sera 5–7 weeks after the onset of COVID symptoms show moderate to strong correlation with virus neutralizing antibody activity in the present patient cohort, however, does not yet support a general use case identifying protected individuals. ELISA tests of multiple manufacturers (following different approaches to include S1, nucleocapsid or RBD antigens) performed similarly in predicting antibody neutralization.

As it seems feasible to improve performance by combining tests against different antigens or optimizing sampling time(s) and assessing different types of immunoglobulins, in special cases (e.g.: vaccination follow-up, plasma screening) and possibly with modified cut-off recommendations, ELISA tests might later be considered as an alternative for cellular neutralization assays.

## Funding

The research was funded by the Higher Education Institutional Excellence Program of the 10.13039/501100005881Ministry of Human Capacities in Hungary, within the framework of the molecular biology thematic program of the 10.13039/501100002332Semmelweis University and of the University of Physical Education. Further funding was provided by the Ministry of Innovation and Technology of Hungary to OrthoSera LLC, provider of plasma therapy to fight COVID-19.

## Availability of data and material

All data generated or analyzed during this study are included in this published article and its supplementary information files, or available from the corresponding author on reasonable request.

## Author statement

Zsófia Szabó: Investigation, Conceptualization, Statistical analysis, Methodology, Data curation, Writing - original draft, review & editing. Tamás G. Szabó: Statistical analysis, Writing - original draft, review & editing. Katalin Kristóf: Investigation, Writing - review & editing. Eszter Barabás: Investigation, Writing - review & editing, Methodology. Barna Vásárhelyi: Resources, Writing - review & editing. Zoltán Prohászka: Conceptualization, Writing - review & editing. Kornélia Bodó: Investigation, Methodology, Data curation. Gábor Kemenesi : Performing virus neutralization assays with human sera, Writing: neutralization methodology, review and editing Fanni Földes: Performing virus neutralization assays with human sera, Writing: neutralization methodology, review and editing. Eszter Fodor: management of subjects and obtaining samples. Ferenc Jakab: Writing: review and editing. Timea Berki : Investigation, Conceptualization, Methodology, review & editing. Zsombor Lacza: conceptualization and management of clinical study, review & editing of manuscript.

## Ethics approval

IRB approval number IV/3457/2-2020-EKU, ClinicalTrials.gov Identifier: NCT04345679.

## Consent to participate and for publication

Informed consent was obtained from all individual participants included in the study. Patients also signed informed consent regarding publishing their data.

## Declaration of competing interest

None.
